# Metallic Nanoparticles in the Food Sector: A Mini-Review

**DOI:** 10.3390/foods11030402

**Published:** 2022-01-30

**Authors:** Cristina Couto, Agostinho Almeida

**Affiliations:** 1 TOXRUN–Toxicology Research Unit, University Institute of Health Sciences, CESPU, CRL, 4585-116 Gandra, Portugal; 2 LAQV/REQUIMTE, Department of Chemical Sciences, Faculty of Pharmacy, University of Porto, Rua de Jorge Viterbo Ferreira 228, 4050-313 Porto, Portugal; aalmeida@ff.up.pt

**Keywords:** metallic nanoparticles (MNPs), food, food packaging, food analysis

## Abstract

Nanomaterials, and in particular metallic nanoparticles (MNPs), have significantly contributed to the production of healthier, safer, and higher-quality foods and food packaging with special properties, such as greater mechanical strength, improved gas barrier capacity, increased water repellency and ability to inhibit microbial contamination, ensuring higher quality and longer product shelf life. MNPs can also be incorporated into chemical and biological sensors, enabling the design of fast and sensitive monitoring devices to assess food quality, from freshness to detection of allergens, food-borne pathogens or toxins. This review summarizes recent developments in the use of MNPs in the field of food science and technology. Additionally, a brief overview of MNP synthesis and characterization techniques is provided, as well as of the toxicity, biosafety and regulatory issues of MNPs in the agricultural, feed and food sectors.

## 1. Introduction

Nanotechnology is the branch of science and engineering that deals with the preparation of nano-size particles, i.e., particles with dimensions from 1–100 nm, using various synthesis strategies that allow obtaining particles with different structures and sizes [1]. With advances in nanotechnology, novel materials (nanomaterials) with peculiar and improved properties (compared to those of atoms, molecules and bulk materials), attributed to their high surface-to-volume ratio (35–45% higher compared to atoms), have progressively emerged [2]. Nanoparticles (NPs) have both solute and separate particle phase properties [3]. The unique extrinsic property of NP of having a large specific surface area is an important feature that contributes to some particular characteristics, namely strong surface reactivity [1,4].

In the last decades, due to their particular properties, NP have received great attention in the most diverse areas, from optical, electronic and medical applications to their use as sensors and catalysts, being used in various fields such as physics, organic and inorganic chemistry, molecular biology, and medicine [1,5,6]. The use of NP in the food sector is relatively more recent, but the special characteristics of mechanical resistance, diffusivity, optical properties and solubility of NP have also led to their introduction in all stages of the food sector chain: production, processing, packaging, transport and storage, with notable impacts on food quality and safety [7,8].

Metallic (or metal) nanoparticles (s) constitute a special and particularly valuable group of NP. Their physicochemical properties depend mainly on the (relatively) free surface electrons, and they present unique characteristics such as high surface energies, high plasmon excitation efficiencies and a variety of unusual and interesting optical properties [9,10]. The use of MNPs in food technology and industry plays a key role in protecting, preserving and extending the shelf life of food [1,10,11,12]. In particular, the use of MNPs has allowed the improvement of food packaging characteristics, such as mechanical properties, permeability to water vapor and antibacterial activity [13,14], allowing not only the maintenance of freshness and increases in the shelf life of products, but even the production of safer and more ecologically sound (degradable) food packaging [8]. Additionally, MNPs allowed the development of new devices, namely nanosensors [13,14], and methods for food analysis [8].

The increasing use of MNPs has naturally raised environmental and ecological concerns. One answer to these concerns is the synthesis of metallic nanocomposites (consisting of several nanomaterials entrapped in a bulk material) starting from natural raw materials. In this synthesis strategy, natural biopolymers (e.g., starch, agar, gelatin, chitosan and cellulose [11]) are combined with one or more different types of MNP, and act not only as vehicles for the particles, but also give rise to hybrid systems with specific characteristics, such as large surface areas, ordered crystalline structures and highly regularized pores [11,15,16]. This new approach has been widely explored in recent years due to its environmental friendliness, easy processing and the particularly suitable mechanical and barrier characteristics of the resulting materials [17].

Metal nanotechnology applied to the food sector is, therefore, a clear example of an emerging technology that can have both positive and negative impacts. Its applications are ubiquitous, from agriculture to food processing, packaging and storage, including laboratory quality control. Naturally, it also raises safety concerns, especially regarding its potential effects on human health. A thorough knowledge of the safety and proper risk assessment of the use of MNPs in food processing and packaging is, therefore, crucial. In this mini-review, we aim to present the latest developments in the use of MNPs in food technology, as well as their impact on food quality and safety.

## 2. Synthesis and Stabilization of MNPs

One of the biggest limitations related to the use of MNPs is the lack of an effective synthesis process that can lead to homogeneous sizes and shapes, as well as particles with little or no toxicity for both humans and the environment [18]. Different physical and chemical methods are generally used for the preparation and stabilization of MNPs, with the reduction of metal ions in a solution using suitable reducing agents being the most widely used chemical process [18]. Precursor concentration, temperature and nature of reducing agent and solvent are the most critical parameters. The kinetics of the interaction between metallic ions and the reducing agent and the kinetics of the adsorption process of the stabilizing agent with the nascent MNP influence their structure, size uniformity and general physicochemical properties [19].

**Figure 1:**
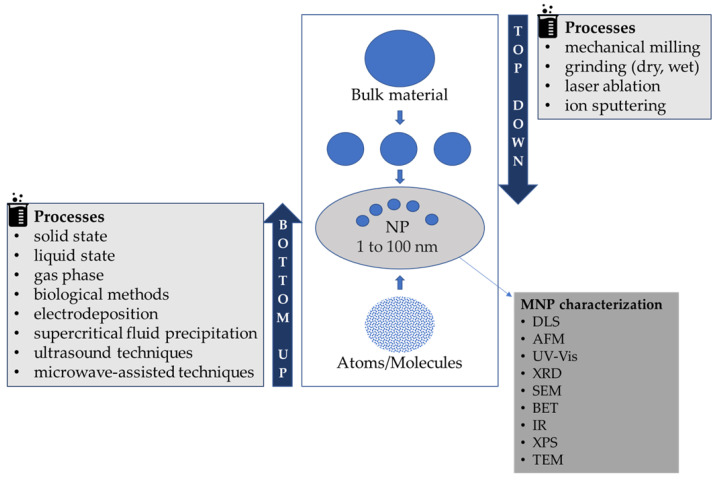
Flowchart representation of MNP preparation and characterization. DLS: dynamic light scattering, AFM: atomic force microscopy, UV-Vis: ultraviolet-visible spectroscopy, XRD: X-ray diffraction, SEM: scanning electron microscopy, BET: Brunauer–Emmett–Teller method, IR: infrared spectroscopy, XPS: X-ray photoelectron spectroscopy, TEM: transmission electron microscopy.

Several approaches are employed for the preparation of MNPs and can generally be classified as the bottom-up and top-down methods (Figure 1), depending on the starting material [3]. In top-down methods, the bulk material is made into nano-sized particles by reducing the size of the starting material through mechanical milling/grinding (dry, wet), laser ablation or ion sputtering. These methods are easy to perform but inadequate for preparing very small-sized, informal shaped particles, and changes in the surface chemistry and physicochemical characteristics of MNPs are observed [3].

The bottom-up approach is based on the formation of MNPs from smaller entities by joining atoms, molecules or small particles. In this approach, the nanostructured building blocks of the nanoparticles are previously formed and then assembled to produce the final MNP [3]. Examples of bottom-up methods are solid state, liquid state, gas phase and biological methods, as well as electrodeposition or supercritical fluid precipitation processes and ultrasound or microwave-assisted techniques [3]. Biological processes to synthesize nanoparticles are considered simpler, cheaper and more ecologically sound compared to conventional chemical methods [18,20]. For this biogenic or green synthesis, fungi, actinomycetes, algae and even higher plants have been used and have shown themselves as potential nanofactories, with high cost-effectiveness and respect for the environment [20]. Nanoparticles produced by biological processes tend to have greater catalytic reactivity and a greater specific surface area due to improved contact between the reducing enzymes and the metal ion, but biological processes are slower, and the size and shape of the formed nanoparticles are more heterogeneous [21].

MNPs are commonly stabilized through the adsorption of high-molecular compounds, which form a dispersant layer around the particle surface and prevent their aggregation/coagulation [22]. These “capping” agents can significantly change the physicochemical and biological characteristics of an MNP [23].

Several methods and techniques are available for evaluating the size and physicochemical properties of MNPs, such as dynamic light scattering (DLS), the Brunauer–Emmett–Teller (BET) method, atomic force microscopy (AFM), infrared and UV-Vis spectrophotometry, X-ray diffraction (XRD) and X-ray photoelectron spectroscopy (XPS), scanning electron microscopy (SEM) and transmission electron microscopy (TEM) [6].

## 3. Uses of MNPs in the Food Sector

**Figure 2:**
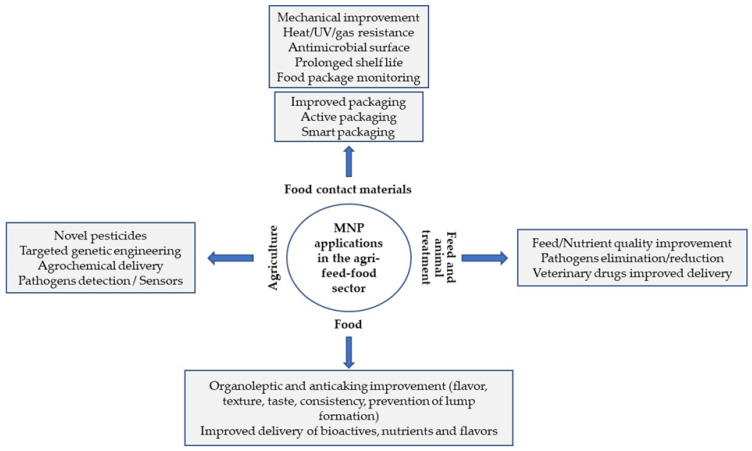
Pictorial representation of some of the main applications of nanotechnology in different agricultural and food sectors (adapted from [25]).

Most applications of MNPs in agriculture and the food industry are focused on improving the organoleptic properties of foods (taste, color, texture), increased nutrient absorption, targeted delivery of nutrients and bioactive compounds, stabilization of active ingredients and antimicrobial action against foodborne pathogens. Many are related to packaging innovations to preserve product quality and improve shelf life. MNPs have also been used as sensors to monitor food quality and safety (Figure 2) [24].

Briefly, MNPs are used to:

- Develop antimicrobial agents with the potential to improve the shelf life of foods and prevent microbial growth. MNPs can destroy microbial cells through different mechanisms and have the potential to inhibit biofilm development [26]. Antimicrobial activity can result from MNP adsorption on the cell wall, destruction of the cell membrane by free radicals, induction of intracellular release of reactive oxygen species (ROS), interaction of metal ions with cell respiratory enzymes and interaction with DNA and proteins [27,28]. This antimicrobial activity depends on the MNP synthesis method, size, shape and type, and nature of the capping agents [23].
- Develop active, smart or biodegradable packaging with increased UV protection and antimicrobial activity, enhanced thermal, hydrophobicity (reduced water vapor permeability) and oxygen barrier properties; the ability to change product color; enhanced radical and oxygen scavenging activities; improved mechanical properties (tensile strength, film thickness and transparency, barrier properties), etc. [8]. Improved food packaging based on functional nanomaterials can be classified into four different categories: physically improved packaging (improved mechanical strength, temperature and moisture stability, gas barrier functions, flexibility and durability); biochemically improved packaging (improved biodegradability, edibility, biocompatibility, low-waste and eco-friendly features); improved packaging with active functions (effect on packaged foods with regard to taste, freshness and shelf life); improved packaging with smart functions (e.g., nanosensors to monitor food conditions such as oxygen levels, freshness and the presence of pathogens) [29].
- Develop MNP-based sensors useful for detecting food contaminants, particularly microbiological pathogens [8,30].

The main current uses of MNPs in the food sector are listed in Table 1.

### 3.1. Gold Nanoparticles (Au-NPs)

Au-NPs have received considerable interest in the food industry (Table 1) due to their potential antibacterial activity, inert and nontoxic nature, and oxidative catalytic properties, even though the antibacterial effects of AuNPs are a controversial topic [141]. However, the use of Au-NPs in the development of sensors for food contaminants has been shown to be the most promising application, due to their good characteristics regarding reactivity, selectivity and sensitivity.

Pissuwan et al. [34] recently reviewed the use of Au-NPs in the detection of foodborne pathogens. Several Au-NP-based procedures were identified, including liquid-phase colorimetric assays, refractometric sensing, surface-enhanced Raman scattering (SERS) and immunochromatographic or electrochemical techniques, among others. Another recent review highlighted the latest advances in Au-NP-based biosensors with a focus on fast, low-cost portable biosensors, allowing for on-site assessment of food safety [142]. Progress specifically in the optical detection of pathogenic bacteria using noble metal nanoparticles, with a focus on colorimetric, SERS and fluorescence assays, was also recently reviewed [143]. Using SERS, a sandwich immunoassay was developed for the detection of *E. coli* using alkaline phosphatase and both spherical gold-coated core-shell Au-NPs and rod-shaped Au-NPs [38]. Additionally, a novel label-free three-dimensional SERS substrate based on black phosphorus-Au filter paper was tested in the rapid detection and discrimination of *S. aureus*, *L. monocytogenes* and *E. coli*, having shown to be a highly sensitive, low-cost alternative to conventional substrates [39].

Other analytical applications of Au-NPs, in addition to food pathogens, have been described. Yang et al. [31] developed an Au-NP-based SERS procedure to improve the detection of bisphenol A residues in milk. Fan et al. [32] developed an electrochemical sensor to determine the Ca^2+^ content in meat. Oxygen plasma-treated graphene was used with Au-NPs. Bhardwaj et al. [37] developed a surface plasmon resonance (SPR)-based nanosensor (chip) with incorporation of Au-NPs for the detection of aflatoxin in wheat samples.

Hybrid metal-polymer matrices, which have distinctive properties as already mentioned, are a new class of materials with applications in food quality control. Mohan et al. [36] tested the effectiveness of nanocomposites containing Au-NPs and chitosan in monitoring the storage conditions of frozen products, where a change in color could indicate an out-of-range temperature.

### 3.2. Silver Nanoparticles (Ag-NPs)

The role of Ag-NPs in food applications has been extensively reviewed [144]. The exact mechanism of action of Ag-NPs on cells is still unknown, but they can physically interact with the cell surface of different bacteria. Detailed discussion on the possible mechanisms of action of MNPs can be found in [145]. The mechanisms include adhesion to the surface of the bacterial cell wall or membrane, penetration into the cell and disruption of intracellular organelles and biomolecules, induction of oxidative stress and modulation of signal transduction pathways. Adhesion and accumulation of Ag-NPs on the cell surface have been particularly noted in Gram-negative bacteria [28,146].

The antimicrobial effects of Ag-NPs are discussed in several papers (Table 1), and to improve them, the combination of different MNPs or their combination with natural bioactive compounds to obtain biomaterials with increased efficacy has also been tried [147,148]. Packaging materials containing Ag-NPs have been shown to significantly extend the shelf life of products such as rice, potatoes, etc. [67]. Chitosan has been widely used as a polymer in these applications due to its biocompatibility, biodegradability and intrinsic antibacterial properties. Kadam et al. [65] used chitosan-based nanocomposite films impregnated with Ag-NPs biologically synthesized with an extract of Nigella sativa. The film showed a pH-dependent sustained release of Ag-NPs and Ag+ and significant antibacterial activity. Ahmed et al. [135] reported the use of poly(lactic acid) (PLA) biopolymers incorporated with Ag-Cu-NPs and cinnamon oil with significant antimicrobial activity against *Salmonella* Typhimurium, *C. jejuni*, and *L. monocytogenes* in inoculated chicken tissues. Another study, using PLA impregnated with Ag-NPs and titanium dioxide, showed good antimicrobial activity against *E. coli* and *L. monocytogenes* [139]. In another study where a PLA film was used to store rice at high temperature and humidity, due to the presence of Ag^+^ ions, the film showed a cidal effect on *A. flavus* and considerably delayed rice aging [67]. A nanocomposite consisting of a polypropylene (PP) matrix impregnated with Ag-NPs was also tested, and its antimicrobial effectiveness was evaluated against *E. coli* and *S. aureus*. There was a significant enhancement in activity compared to that from isolated, conventional Ag-NPs [53]. In another study [60], a novel trinary nanocomposite film based on tragacanth, hydroxypropylmethylcellulose and beeswax impregnated with Ag-NPs was developed. It proved to be suitable for use in food packaging, as it showed a dose-dependent inhibitory effect against *B. cereus*, *S. aureus*, *S. pneumoniae*, *L. monocytogenes*, *E. coli*, *K. pneumoniae*, *P. aeruginosa*, and *Salmonella* Typhimurium. Olmos et al. [55] studied the effectiveness of low-density polyethylene-Ag nanocomposites in preserving food against biofilm-forming *E. coli*, and they proved useful for both storage and general-purpose containers. Other hybrid materials, such as polyvinylalcohol (PVA)/nanocellulose/Ag nanocomposite films, were also tested in packaging applications to protect against methicillin-resistant *S. aureus* and *E. coli* [30]. Blend films of PVA-montmorillonite K10 clay nanocomposite with in situ-generated Ag-NPs (using a ginger extract-mediated synthesis procedure) were used to manufacture biodegradable pouches that showed ability to prevent microbial spoilage of chicken sausages [62]. Other studies reported the development of hybrid nanomaterials obtained by one-pot synthesis of Ag-NPs, CuO-NPs and ZnO-NPs during the regeneration of cellulose from cotton linters and microcrystalline cellulose [63]. Vishnuvarthanan et al. [66] developed a carrageenan/Ag-NP/laponite nanocomposite coated on oxygen plasma surface–modified PP film. Ag-NPs were synthesized using extract of *Digitalis purpurea*. Characteristics such as adhesion, mechanical barrier and antimicrobial properties of the nanocomposites were significantly increased, as well as the activity against *E. coli* and *S. aureus*. Dairi et al. [64] studied nanobiocomposite films of Ag-NPs, gelatin-modified montmorillonite nanofiller and thymol, biogenically synthesized using *Curcuma longa* tuber extracts. The films showed antibacterial, antifungal and antioxidant activities, with the ability to extend the shelf life of fruits. Starch (St)-based nanocomposite films containing single or combined Ag, ZnO and CuO nanoparticles were prepared, and microbial tests showed that St-Ag and St-CuO films had the highest antibacterial activity against *E. coli* and *S. aureus*. Increasing the NP concentration from 1 to 3% (*w*/*w*) increased the antibacterial effect. The combined use of Ag/ZnO/ CuO-NPs in the formulation showed a synergistic effect on the antimicrobial and mechanical properties of the films, allowing the dose reduction of each individual MNP [126].

Ag-NPs have also been shown to be very efficient in detecting food spoilage and evaluating post-harvest spoilage of agricultural and horticultural crops. One work described the use of cysteine- and histidine-incorporated Ag-NPs to detect lactic acid in fresh milk by color change [75]. A recent review describes other Ag-NP films that have been applied to various fruit crops such as bananas, tomatoes and kiwis to detect spoilage through color change [13].

### 3.3. Copper Nanoparticles (Cu-NPs)

Cu-based nanocomposites are particularly interesting because they are less expensive than Ag equivalents. They have been reported to have potential antibacterial activity against a wide range of Gram-positive and Gram-negative bacteria due to Cu ion release, Cu-NP release from nanocomposites, and inhibition of bacterial biofilm formation [11]. A review on the use of Cu-polymer nanocomposites presented several studies showing antimicrobial activity against *S. aureus*, *E. coli*, *S. cerevisiae*, *Streptococcus* spp., and *Pseudomonas* spp. in food [76]. Antimicrobial activity against *S. aureus*, *S. epidermidis*, *B. cereus*, *E. coli*, *E. faecalis*, *Salmonella* spp. and *P. aeruginosa* in the packaging and preservation of food products such as meat was evaluated using a biodegradable hydroxypropylmethylcellulose matrix impregnated with Cu-NPs [77]. Varaprasad et al. [125] produced metal-oxide polymer nanocomposite films using biodegradable poly-ε-caprolactone, polyethylene terephthalate (PET; discarded oil bottles) and ZnO-CuO nanoparticles. Metal oxide-polymer nanocomposite films have demonstrated excellent mechanical properties and have been reported to be functional in household packaging. Works by Arfat et al. [137,138] evaluated Ag-Cu alloy nanoparticle composite films based on agar or guar gum. In addition to showing good mechanical strength, UV light protection and oxygen barrier efficacy, the films showed antibacterial activity against *L. monocytogenes* and *Salmonella* Typhimurium, proving to have high potential as films usable in active food packaging. Ahmed et al. [136] prepared plasticized PLA-based nanocomposite films incorporating polyethylene glycol and Ag-Cu alloy or ZnO nanoparticles. Later, the same authors prepared plasticized PLA composite films impregnated with Ag-Cu-NPs and cinnamon essential oil, as already mentioned. Bacterial growth was remarkably reduced when a film containing 50% cinnamon essential oil was used in the packaging of chicken meat [135]. Active biodegradable poly(3-hydroxybutyrate-co-3-hydroxyvalerate) melt mixed nanocomposites and bilayer structures containing CuO-NPs were developed and characterized by Castro Mayorga et al. [82]. The products exhibited significant bactericidal and virucidal action against foodborne pathogens *S. enterica*, *L. monocytogenes* and murine norovirus.

### 3.4. Zinc Nanoparticles (Zn-NPs)

Transition metal oxide nanomaterials have shown high antibacterial activity and, among them, ZnO-NPs showed clear superiority. This is due to a distinctive electronic configuration, which, in particular, causes them to lead to the formation of ROS following light absorption [149].

The mechanisms responsible for the antimicrobial activity of ZnO-NPs are not fully understood, but it is believed that it will depend on the action of released Zn^2+^ ions (with antimicrobial activity) [145,149] and that the contact of the nanoparticles with the bacterial surface is capable of causing the formation of electrostatic forces that damage the cell membrane [150]. However, the most plausible mechanism is the formation of reactive oxygen species, including hydrogen peroxide, although it is not entirely clear how these species are produced [28,145,149,150,151,152].

Different polymers such as chitosan, poly(3-hydroxybutyrate), poly(butylene adipate-co-terephthalate), low-density polyethylene, semolina flour and bovine skin gelatin have been used to produce ZnO-NP-based nanocomposites, and it has been shown that the incorporation of ZnO-NPs in polymeric films makes them fire resistant, lighter, thermally and mechanically improved and less permeable to moisture and gases, thus more suitable for food packaging [15]. Marra et al. [84] proved that a PLA film with 5% (*w*/*w*) ZnO is suitable for use in food packaging, having good tensile properties, lower permeability to O_2_ and CO_2_ and excellent antimicrobial activity against *E. coli.* ZnO nanoparticles were used by Zhang et al. [85] to develop a paper-based packaging material coated with a ZnO-PLA layer, and it showed efficacy in inactivating both *E. coli* and *S. aureus*.

A study described the production of sustainable, low-cost, antimicrobially active bio-nanocomposites consisting of ZnO-NPs incorporated in chitosan, obtained from food industry by-products (apple peel). Their hydrophobic, mechanical, optical and barrier properties were characterized. The films proved to be efficient in extending the shelf-life of fresh poultry meat, and the incorporation of ZnO-NPs enhanced their antimicrobial and antioxidant properties [86]. A gelatin/ZnO-NP nanocomposite film was evaluated, and its antibacterial activity was confirmed, showing it to be more active against Gram-positive than Gram-negative foodborne pathogenic bacteria [87]. In a work by Arfat et al. [83], a nanocomposite film consisting of fish protein isolate plus fish skin gelatin and ZnO-NPs was characterized, and also exhibited strong antibacterial activity. A nanocomposite film consisting of chitosan-ZnO-cellulose containing nisin revealed potent antibacterial activity and could be used for cheese packaging [88]. Nanocellulose has proven to be a promising natural material for the production of hybrid nanocellulose-ZnO-NP-based composites with excellent mechanical, UV protection and antibacterial properties, suitable for use in food packaging [89]. Another study explored the natural properties of chitin combined with ZnO/Ag-NPs incorporated into carboxymethylcellulose, and the hybrid material allowed obtaining films with antibacterial activity against both Gram-positive and Gram-negative bacteria [127]. The incorporation of ZnO-Ag, with *Thymus vulgaris* leaf extract as a stabilizer, into poly(3- hydroxybutyrate-co-3-hydroxyvalerate)-chitosan yielded a novel biodegradable biopolymer nanocomposite. It presented good mechanical characteristics and strong antimicrobial activity, which allowed an improvement in the shelf life of poultry meat, suggesting a potential for replacing the traditional petrochemical-based polymers currently used [128].

The antimicrobial properties of chitosan-ZnO nanocomposite coatings on polyethylene (PE) films were studied, and the complete inactivation of food pathogens *S. enterica*, *E. coli* and *S. aureus* was observed [90]. Chitosan-ZnO nanocomposite portable pouches were developed as smart packaging. A one-pot procedure was adopted for the preparation of the films, which exhibited excellent antimicrobial activity in the packaging of raw meat [92].

### 3.5. Titanium Nanoparticles (Ti-NPs)

Titanium nanostructures have also shown promise with regard to food processing and safety due to their low cost, chemical stability, photocatalytic activity and biocompatibility, in addition to their antimicrobial activity [99]. The antimicrobial activity is probably due to the generation of reactive oxygen species (ROS) [99,145]. Recently, most attention has focused on the combination of TiO_2_-NP and chitosan. Youssef et al. [153] used a chitosan/PVA/TiO_2_-NP nanocomposite as packaging material for soft white cheese, achieving improved shelf life and reduced bacteria, yeast and mold counts compared to the control. Another chitosan film was prepared with *Cymbopogon citratus* essential oil (CCEO) and TiO_2_-NPs, and minced meat was packaged with the developed films and stored at 4 °C for 10 days. The incorporation of TiO_2_-NPs increased the water vapor permeability and the tensile strength, while the addition of CCEO extended the meat shelf life [101]. Another report described the effect of a gelatin/agar bilayer film and nanocomposites containing different concentrations of TiO_2_-NPs on the oxidative stability of fish oil. Their ultraviolet and oxygen barrier properties allowed minimum photo-oxidation and auto-oxidation during the storage period [102]. A recent review focused on PLA/TiO_2_ composites, detailing the approaches used to increase the TiO_2_ dispersion and properties of the composites, and concluded that they are promising materials for food packaging applications [106]. A study in which TiO_2_-NPs were added to PLA matrices showed good results against a wide variety of bacterial strains [103]. Concerns associated with migration from PLA/TiO_2_ and PLA/TiO_2_/Ag composite films were the aim of a study using cottage cheese samples, and their safety as antimicrobial food packaging films was confirmed [140].

The effectiveness of edible nanocomposite films consisting of whey protein isolate/cellulose nanofiber containing TiO_2_ and a rosemary essential oil in preserving the microbial and sensory quality of lamb meat during storage at 4 °C was evaluated, and greater inhibition was observed against Gram-positive versus Gram-negative bacteria [105]. A PE-based nanocomposite packaging material containing attapulgite and Ag, SiO_2_ and TiO_2_ (200–400 nm) particles was developed and tested in the preservation of mushrooms (*Flammulina velutipes*) during storage at 4 ºC. It showed the ability to regulate oxygen and carbon dioxide levels, eliminate released ethylene (possibly absorbed by nanoparticles) and inhibit microbial growth, proving superiority over normal PE material [154]. The migration of ethylene glycol from PET bottles (neat PET and PET nanocomposites) was investigated in a study using TiO_2_-NPs [108]. The migration test (acidic food simulant) showed a lower release of ethylene glycol from nanocomposite bottles (0.09 mg/kg after 15 days versus 0.16 mg/kg after 15 days in neat PET bottles).

### 3.6. Other MNPs

There are some reports on the use of other metals and metal oxide nanoparticles in active food packaging applications and analysis: for example, oxygen scavenging films based on poly(3-hydroxybutyrate) (PHB) impregnated with Pd-NPs [119] and radical scavenging films containing Se-NPs impregnated in a multi-layer plastic packaging material, which extended the shelf life of food products (hazelnuts, walnuts and potato chips), showing antioxidant properties [121]. Iron NPs and especially magnetic Fe-NPs prepared using iron oxides such as magnetite (Fe_3_O_4_) and its oxidized form, maghemite, have also been used [155]. Magnetic Fe-NPs have been used as colorants and sources of bioavailable iron. Many works report the combination of FeO-NPs with carbon nanotubes [123] or other matrices as extraction sorbents in food analysis [156,157]. Magnetic MNPs have been used for the analysis of inorganic and organic compounds in milk, fruit, oil, cereal-based products, beverages, eggs, cacao, and honey, among others [155,156,157].

## 4. Safety and Regulatory Issues

New products and ingredients can pose risks to both human health and the environment. The potential toxicity and the biosafety of MNPs and MNP-containing materials is a major concern in the agricultural and food sectors. Polymers commonly used as films are predominantly composed of environmentally friendly and biocompatible materials. However, MNPs are often considered to have toxic potential, depending on exposure factors. Their possible toxicological effects have been extensively studied by both in vitro and in vivo approaches. Kumar et al. [158] reviewed the toxicological effects of MNPs in different experimental models, such as bacteria, microalgae, zebrafish, crustaceans, fish, rat, mouse, pig, guinea pig, human cell lines, and humans. The effects on organs (liver, kidney, spleen, sperm, neural tissues, liver lysosomes, spleen macrophages, glioblastoma cells, hematoma cells and various mammalian cell lines) were also evaluated. Several studies showed that Ag-NPs induced genotoxicity and cytotoxicity in fish, accumulation in gill tissues, lysosomal destabilization in adults and adverse effects on oyster embryonic development, oxidative stress and p53 protein expression, and induction of apoptosis and oxidative stress in the liver in zebrafish [159,160,161]. ZnO-NP toxicity has been reported in human cervix carcinoma (HEp-2), human HEK293 hepatocytes, and human bronchial epithelial cell lines, demonstrating that ZnO-NPs were toxic to cells, causing DNA damage, oxidative stress and reduction in cell viability [162]. The toxicity of Ti-NPs was studied by Kiss et al. [163], and they were shown to affect cell differentiation, proliferation, apoptosis and mobility. Bour et al. [164] demonstrated the toxicity of CeO_2_-NPs to aquatic environments by inhibiting living systems of different trophic levels. Rajput et al. [165], addressing an important ecotoxicological concern, investigated the phytotoxicity of CuO-NPs in spring barley, one of the most important staple food crops, studying their effects on *H. sativum* grown in a hydroponic system.

It must be stressed that the recommendation of the Organisation for Economic Cooperation and Development (OECD) Council on the Safety Testing and Assessment of Manufactured Nanomaterials (adopted on 19 September 2013) assumes “that the approaches for the testing and assessment of traditional chemicals are in general appropriate for assessing the safety of nanomaterials, but may have to be adapted to the specificities of nanomaterials” [166]. Thus, regulation of nanomaterials and nanotechnology is of utmost importance. In particular, it is imperative to regulate the production, handling and use of nanoparticles and nanomaterials, either by legislation or simply by guidelines and recommendations [167,168]. A critical review on the migration potential of nanoparticles in food contact plastics was provided by [169].

The European legislation most closely related to the use of MNPs in the food sector is shown in Table 2.

Regulation (EC) No 178/2002 (General Food Law Regulation) is the foundation of food and feed law in the European Union (EU). It established the general framework for the development of legislation both at EU and national levels, laying down the general principles and requirements of food law, and the general procedures in matters of food safety, and covers all sectors of the food chain “from farm to fork” (including feed production, primary production, food processing, storage, transport and retail sale). It also established the European Food Safety Authority (EFSA), an agency responsible for scientific advice and support, and created the system for the management of emergencies and crises, including the Rapid Alert System for Food and Feed (RASFF) [170].

“Nanofoods”—comprising “food that has been cultivated, produced, processed or packaged using nanotechnology techniques or tools, or to which manufactured nanomaterials have been added” [171]—are covered by Regulation (EU) 2015/2283 on novel foods. Under this Regulation, food consisting of “engineered nanomaterials” should be considered a novel food, and for the purposes of the Regulation, “engineered nanomaterial” means “any intentionally produced material that has one or more dimensions of the order of 100 nm or less or that is composed of discrete functional parts, either internally or at the surface, many of which have one or more dimensions of the order of 100 nm or less, including structures, agglomerates or aggregates, which may have a size above the order of 100 nm but retain properties that are characteristic of the nanoscale” [172].

Regulation (EU) No 1169/2011, on the provision of food information to consumers, lays down that “all ingredients present in the form of engineered nanomaterials shall be clearly indicated in the list of ingredients” (with the word “nano” in brackets after the name of the ingredient) [173].

The Food Additives Regulation (Regulation (EC) No 1333/2008) stipulates that when there is a “significant change”, namely “a change in particle size, for example through nanotechnology”, in an existing food additive, it shall be considered a new additive and must be submitted for evaluation by the EFSA [174]. The same applies to substances under Regulation (EU) No 609/2013 on food intended for infants and young children, food for special medical purposes, and total diet replacement for weight control [175].

Commission Regulation (EU) No 10/2011 on plastic materials and articles intended to come into contact with food highlights that nanoparticles (“substances in nanoform”) may present different toxicological properties than the same substance in conventional particle size. Therefore, they must necessarily be subject to a specific risk assessment and must only be used after explicit authorization [176]. Idem for Commission Regulation (EC) No 450/2009 covers active and intelligent materials and articles intended to come into contact with food (defined as “materials and articles which monitor the condition of packaged food or the environment surrounding the food”) [177].

Very recently (August 2021), the EFSA released two updated guidelines—“Guidance on risk assessment of nanomaterials to be applied in the food and feed chain: human and animal health” [178] and “Guidance on technical requirements for regulated food and feed product applications to establish the presence of small particles including nanoparticles” [179]—applicable in the regulated areas of food and feed products (novel foods, food contact materials, food/feed additives, etc.). The first guidance focuses specifically on physicochemical characterization, key parameters to be evaluated, methods and techniques that can be used for the characterization of nanomaterials and their determination in complex matrices, as well as aspects related to exposure assessment and hazard identification and characterization [178]. The second guidance describes the assessment criteria (including solubility and dissolution rates of nanoparticles) for deciding when conventional risk assessment should be complemented with nano-specific considerations [179].

In the U.S., the Food and Drug Administration (FDA) has issued a Guidance for Industry titled “Considering whether an FDA-regulated product involves the application of nanotechnology” [180], which represents the Agency’s current thinking on this topic. It applies to all “FDA-regulated products”, which includes food substances (including food for animals) and dietary supplements.

In another Guidance for Industry, titled “Assessing the effects of significant manufacturing process changes, including emerging technologies, on the safety and regulatory status of food ingredients and food contact substances, including food ingredients that are color additives” [181], additional guidance is provided regarding food ingredients and food contact substances.

Briefly, FDA regulatory intervention regarding applications of nanotechnology or the use of nanomaterials involves two different approaches [182]:

Where products are subject to mandatory premarket review (e.g., food additives, certain new ingredients in dietary supplements), applicants are required to submit data on product safety. Moreover, the premarket review procedure includes special attention to whether the use of nanomaterials suggests the need for additional data on safety.

Where such mandatory premarket review does not exist (e.g., dietary supplements, food), manufacturers are encouraged to consult with the Agency before bringing their products to market to assess the risks of unintended harm to human or animal health.

## 5. Final Remarks

MNPs have shown great potential in the food sector, contributing to enhance organoleptic properties and improve packaging and storage conditions for food products through innovative, active and intelligent packaging materials, contributing to increase safety, preserve nutritional value and extend product shelf life. In particular, there has been enormous progress in the development of composite nanomaterials with increased biocompatibility through the use of natural biopolymers and the use of more environmentally friendly synthesis processes.

However, the potential benefits and risks must to be carefully assessed. Strategies for specific biosafety risk assessment and definition of regulatory frameworks are still a challenge that requires extensive research. In particular, innovative tests are needed to assess the potential long-term effects of MNPs on human health and the environment. Furthermore, it is essential to formulate strict regulations regarding their safe use in the food industry and to closely monitor compliance.

## Figures and Tables

**Table 1:** Main uses of metallic nanoparticles (MNPs) in the food sector.

MNP	Application	Film/Analyte/Food	Ref.
**Au-NP**	SERS sensor	Bisphenol/milk	[31]
	Electrochemical sensor	Ca^2+^/meat	[32]
	Electrochemical biosensor	Bisphenol A/waters	[33]
	Rapid detection of single or multiple foodborne pathogens	Foodborne pathogens	[34]
	SPR with Au-NPs	−	[35]
	Temperature indicator	Chitosan-capped Au-NPs/frozen products	[36]
	SPR biosensor	Aflatoxin B1	[37]
	SERS active sandwich immunoassay	*E. coli*	[38]
	Filter paper-based SERS substrates	Black phosphorus-Au/*S. aureus*, *L. monocytogenes* and *E. coli*	[39]
	Isothermal RPA detection	*Salmonella* detection/milk	[40]
	Colorimetric sensor	*hlyA* gene and genomic DNA of *Listeria monocytogenes*	[41]
	Aptasensor	*C. jejuni* and *C. coli*/chicken carcass	[42]
	Polymerase chain assay colorimetric sensor	Emetic *Bacillus cereus*/milk	[43]
	Portable plasmonic biosensor	Melamine/infant formula	[44]
	Colloidal Au immunochromatographic strip	Simultaneous detection of *S. boydii* + *E. coli*	[45]
	Paper sensor	*Listeria* spp./milk	[46]
	SERS-based aptasensor	Kanamycin residue/milk	[47]
	Immunochromatographic sensor	26 sulfonamides/commercial honey	[48]
	Low-fouling SPR biosensor	*E. coli* and *Salmonella* spp./cucumber and hamburger	[49]
	Cuvette-type localized SPR optical biosensor	Melamine/infant formulas	[50]
	POC biosensors	Food allergens	[51]
	Optical sensor	Biogenic amines/poultry meat	[52]
**Ag-NP**	Films with enhanced antibacterial and migration properties	PP-Ag nanocomposite	[53]
	Biodegradable food packaging	Chitosan/gelatin/Ag-NP composites/carrot pieces	[54]
	Antimicrobial films	LDPE/Ag-NPs	[55]
	Edible coatings (thin layers of material on the product surface)	Ag-chitosan nanocomposites into chitosan coatings/fresh-cut melon	[56]
	Combined use of gamma irradiation and PE/Ag-NP films	PP/Ag-NPs/fresh bottom mushroom	[57]
	Multifunctional packaging	Chitosan coated PE films (lecithin-liposomes/laurel essential oil/Ag-NPs)/pork	[58]
	Active nanocomposite packaging film	PLA/Ag-NPs/strawberries	[59]
	Physico-mechanical and antimicrobial edible films	Tragacanth/HPMC/bees-wax/Ag-NPs	[60]
	Coating films	Guar gum-Ag coatings/coated kinnow (*Citrus reticulata* cv. Blanco)	[61]
	Antimicrobial food packaging	PVA/nanocellulose/Ag nanocomposite	[30]
	Packaging	Biodegradable PVA-montmorillonite K10 clay nanocomposite blend films with in situ generated ginger extract mediated Ag-NP pouches/chicken sausages	[62]
	Antimicrobial materials	Hybrid nanomaterials(cellulose/Ag-NPs)	[63]
	Active food packaging: antimicrobial/antioxidant	Cellulose acetate/AgNP-organoclay and/or thymol nanobiocomposite films	[64]
	Packaging	Chitosan based nanocomposite films incorporated with biogenic Ag-NPs	[65]
	Packaging	Carrageenan/Ag-NP/laponite nanocomposite coated on oxygen plasma surface–modified PP film	[66]
	Packaging	*A. flavus*/mildew and storage of rice	[67]
	Packaging	Nano-cellulose composite films/grape seed extracts/Ag-NPs (antimicrobial activity against *E. coli* and *S. aureus* + strong antioxidant activity)	[68]
	Packaging	Cellulosic packets impregnated with Ag-NPs/*Aeromonas* sp. isolated from rotten vegetables (tomatoes and cabbage)	[69]
	Colorimetric assay based on Ag-NPs	Melamine/milk	[70]
	Colorimetric sensor	Ag-NP solution synthesized using culture supernatant *B. subtilis*/volatile compounds released during the deterioration of *Musa acuminata* (banana)	[71]
	Colorimetric sensor	Ag-based nanomaterial/onion postharvest spoilage	[72]
	Packaging	Chitosan-Ag-NPs/minced meat	[73]
	Packaging	Agar film containing nanoAg conjugate (*Cymbopogon citratus* extract/nisin/Ag)/*L. monocytogenes*, *S. aureus*, *P. fluorescens*, *A. niger* and *F. moniliforme*	[74]
	Food spoilage detection and post-harvest spoilage	Cysteine and histidine incorporated Ag-NP lactic acid/fresh milk	[75]
**Cu-NP**	Antibacterial surfaces	Antibacterial effect of Cu-polymer nanocomposites	[76]
	Packaging	Biodegradable HPMC matrix/Cu-NPs/ *S. aureus*, *S. epidermidis*, *B. cereus*, *E. coli*, *E. faecalis*, *Salmonella spp.*, *P. aeruginosa*/meat	[77]
	Packaging	Chitosan/soy protein isolate nanocomposite film	[78]
	Amperometric paper sensor	Carbohydrates/soft drinks	[79]
	Electrochemical biosensor	Organophosphorus pesticides (chlorpyrifos, fenthion and methylparathion)/cabbage and spinach extract	[80]
	Electrochemical sensor	Malathion/vegetable extracts	[81]
	Active biodegradable film	Poly(3-hydroxybutyrate-co-3-hydroxyvalerate) nanocomposites/CuO-NPs/ *S. enterica*, *L. monocytogenes* and murine norovirus	[82]
**Zn-NP**	Active food packaging material	Fish protein isolate and fish skin gelatin/ZnO-NPs	[83]
	Packaging	PLA/ZnO biocomposite films	[84]
	Antimicrobial packaging	PLA/ZnO nanocomposite coated paper	[85]
	Biodegradable film	Bionanocomposites of chitosan/ZnO-NPs/apple peels, fresh poultry meat	[86]
	Packaging	Gelatin/ZnO-NP nanocomposite film/ foodborne pathogenic bacteria	[87]
	Packaging	Nanocomposite film of chitosan-ZnO-cellulose/nisin/cheese	[88]
	Packaging	Hybrid nanocellulose-ZnO-NP-based composites	[89]
	Antimicrobial packaging	Chitosan/ZnO coated on PE film	[90]
	Biodegradable food packaging	Soybean protein/ZnO film	[91]
	Food packaging	Chitosan/ZnO antimicrobial pouches/raw meat	[92]
	Food packaging	Ziziphora clinopodioides essential oil/apple peel extract/ZnO-NPs/*Listeria monocytogenes*/sauced silver carp fillet	[93]
	Food packaging	ZnO nanorods/clove essential oil/Type B gelatin composite films/*L. monocytogenes* + *Salmonella* Typhimurium/shrimps	[94]
	Intelligent and active films	Cellulose modified with polypyrrole/ZnO/microbial load chicken thigh	[95]
	Multifunctional bionanocomposite films	Konjac glucomannan/chitosan/ZnO/mulberry anthocyanin extract/*E. coli* and *S. aureus*	[96]
	Coating	Chitosan/ZnO-NPs/microbial growth on fresh-cut papaya	[97]
**Ti-NP**	Active and smart packaging	Chitosan-TiO_2_ composite film/antimicrobial activity against *E. coli*, *S. aureus*, *C.albicans*, *A. niger*/red grapes	[98]
	Coating film	TiO_2_-NPs/chitosan	[99]
	Edible films and coatings	Whey protein nanofibrils/glycerol/TiO_2_/ *L. monocytogenes*, *S. aureus*, *S. enteritidis*, *E. coli/meat*	[100]
	Packaging	Chitosan film/Cymbopogon citratus essential oil/TiO_2_-NPs/minced meat	[101]
	UV absorbent film	Gelatin/agar bilayer film and nanocomposites/TiO_2_-NPs/fish oil	[102]
	Packaging	TiO_2_-NPs/PLA/several bacteria strains	[103]
	Active and smart packaging	PAN/TiO_2_ nanofibers/tomato fruit-ripening test	[104]
	Film	Whey protein/cellulose nanofiber/nanocomposite films/TiO_2_/rosemary essential oil/foodborne bacteria/lamb meat	[105]
	Food packaging	PLA/TiO_2_ composites	[106]
	Amperometric sensor	Graphene/TiO_2_ nanocomposite/hypoxanthine/meat freshness evaluation	[107]
	Packaging	PET/TiO_2_-NPs/ethylene glycol migration	[108]
	Active and smart packaging	PAN/nanofibers/postharvest ripening of bananas	[109]
	Active food packaging (ethylene scavenging + antimicrobial activity)	Chitosan/TiO_2_ nanocomposite film/*S. aureus*, *E.coli*, *Salmonella* Typhimurium, *P. aeruginosa*, *Aspergillus* and *Penicillium*	[110]
	Biodegradable food packaging	Starch/TiO_2_ bionanocomposite	[111]
	Biodegradable food packaging	Chitosan/PVA/Ti-NPs/olive oils	[112]
	Active packaging	Chitosan- TiO_2_ nanocomposite film/tomato storage shelf life	[113]
	Packaging	Chitosan/PVA/skimmed milk acid coagulated cheese (Karish)	[114]
	Edible coating	CMC/gum arabic/gelatin/garlic extract/TiO_2_-NPs/Nile tilapia fish fillets	[115]
**Other single metal MNPs**	Optical biosensor	Single-layer MnO_2_ nanosheets/ascorbic acid/fresh oranges and orange juice	[116]
	Packaging	Chitosan nanocomposite thin films/MgO	[117]
	Active and smart packaging	Nanoscale O_2_ scavengers (Fe particles)	[118]
	Active and smart packaging	O_2_ scavenging films made of PHB/Pd-NPs	[119]
	Active and smart packaging	Nano zeolite-Mo_4_^2−^/avocado ripeness indicator	[120]
	Flexible antioxidant packaging (radical scavenging ability)	Embedded Se-NPs/multi-layer plastic/preservation of packages/hazelnuts, walnuts, potato chips	[121]
	Electrochemical biosensor	Graphene-based electrode/nano/microstructured Pt-NPs/phosphonate organophosphates	[122]
	Magnetic solid phase extraction HPLC-DAD	Fe_3_O_4_ magnetic NPs/multi-walled carbon nanotubes and nanodiamonds/vit.B12/milk-based infant formula, orange and peach juice, meat, salami, powder milk	[123]
	Magnetic solid phase extraction FAAS	Fe_3_O_4_ -sodium dodecyl sulfate-carbazone/ Cd/green tea. Lettuce, ginseng, rice, spice and carrot	[124]
**Mixed** **(bi-/ternary) MNPs**	Nanocomposite films	ZnO/CuO-NPs/poly-ε-caprolactone/terephthalic acid	[125]
	Packaging	Hybrid nanomaterials: Ag-NPs, CuO-NPs, ZnO-NPs/cellulose regenerated from cotton linter and microcrystalline cellulose	[63]
	Packaging	Starch-based nanocomposite films: single or combined Ag, ZnO and CuO-NPs/*E. coli* and *S. aureus*	[126]
	Packaging films	Chitin/ZnO/Ag-NPs/CMC/Gram(+) and Gram(−) bacteria	[127]
	Degradable biopolymer nanocomposite	ZnO/Ag nanocomposite/Thymus vulgaris leaf extract/PHB-co-3-hydroxyvalerate)-chitosan/poultries	[128]
	Films	Furcellaran/gelatin/Se-Ag-NPs/*S. aureus,* Multi Resistant *S. aureus* and *E. coli/* kiwi (*Actinidia arguta*) storage	[129]
	Active packaging	Combination of high-pressure treatment, steak margination and MNPs LDPE/Ag and ZnO-NPs/beef color and shear stress	[130]
	Film	SiO_2_/carbon/Ag ternary hybrid polymeric composite/*S. enteritidis*	[131]
	Packaging films	PLA/bergamot essential oils (BEO) or PLA/BEO/nano-TiO_2_ or PLA/BEO/nano-TiO_2_ + nano-Ag/mangoes	[132]
	Packaging films	LDPE incorporating Ag/CuO/ZnO-NPs/ coliform/ultra-filtrated cheese	[133]
	Electrochemiluminescent immunoassay	Au nanorods functionalized graphene oxide and Pd/Au core-shell nanocrystallines/tomato and chili sauce and powder	[134]
	Active food packaging	PLA films/Ag/Cu-NPs/cinnamon essential oil/chicken meat	[135]
	Nanocomposite films	Ag/Cu and ZnO reinforced PLA	[136]
	Nanocomposite films	Ag/Cu agar-based/*L. monocytogenes*, *Salmonella enterica* serovar Typhimurium	[137]
	Active food packaging material	Ag/Cu guar gum nanocomposite films	[138]
	Packaging film	Ag/TiO_2_/PLA/*E. coli, L. monocytogenes*	[139]
	Packaging	PLA/TiO_2_ and PLA/TiO_2_/Ag composite films/cottage cheese samples	[140]

SERS: surface-enhanced Raman spectroscopy; SPR: surface plasmon resonance; RPA: recombinase polymerase amplification; POC: Point of care; PP: polypropylene; LDPE: low-density polyethylene; PE: polyethylene; PLA: polylactic acid; HPMC: hydroxypropylmethylcellulose; PVA: polyvinylalcohol; PAN: polyacrylonitrile; PET: polyethylene terephthalate; CMC: carboxymethyl cellulose; PHB: poly-3-hydroxybutyrate.

**Table 2:** European Union legislation relevant to the use of metallic nanoparticles (MNPs) in the food sector.

Law	Aim and Scope
Regulation (EC) No 178/2002 (General Food Law Regulation)	Laying down the general principles and requirements of food law, establishing the European Food Safety Authority and laying down procedures in matters of food safety
Regulation (EU) 2015/2283 (Novel Foods)	Lays down rules for the placing of novel foods on the market within the Union
Regulation (EC) No 1333/2008	Community lists of approved food additives, conditions of use of food additives in foods, and rules on the labelling of food additives
Regulation (EU) No 609/2013	On food intended for infants and young children, food for special medical purposes, and total diet replacements for weight control
Regulation (EC) No 1334/2008	On flavourings and certain food ingredients with flavouring properties
Regulation (EC) No 1332/2008	On food enzymes
Directive 2002/46/EC	On the approximation of the laws of the Member States relating to food supplements
Regulation (EC) No 1925/2006	On the addition of vitamins and minerals and of certain other substances to foods
Regulation (EC) No 1935/2004	On materials and articles intended to come into contact with food
Commission Regulation (EU) No 10/2011	On plastic materials and articles intended to come into contact with food
Commission Regulation (EC) No 450/2009	On active and intelligent materials and articles intended to come into contact with food
Regulation (EU) No 1169/2011	On the provision of food information to consumers
Regulation (EC) No 1924/2006	On nutrition and health claims made on foods

## Data Availability

Not applicable.
